# Electrospun Fibers from Biobased and Recycled Materials for Indoor Air Quality Enhancement

**DOI:** 10.3390/molecules30061214

**Published:** 2025-03-08

**Authors:** Natalia Czerwinska, Chiara Giosuè, Nicola Generosi, Mattia Pierpaoli, Rida Jbr, Francesca Luzi, Valeria Corinaldesi, Maria Letizia Ruello

**Affiliations:** 1 Department of Science and Engineering of Matter, Environment and Urban Planning (SIMAU), Università Politecnica delle Marche, INSTM Research Unit, 60131 Ancona, Italy; n.czerwinska@staff.univpm.it (N.C.); n.generosi@staff.univpm.it (N.G.); f.luzi@staff.univpm.it (F.L.); v.corinaldesi@staff.univpm.it (V.C.); m.l.ruello@univpm.it (M.L.R.); 2 Department of Metrology and Optoelectronics, Faculty of Electronics, Telecommunication and Informatics, Gdańsk University of Technology, 80-233 Gdańsk, Poland; mattia.pierpaoli@pg.edu.pl; 3 Department of Civil, Environmental and Mechanical Engineering, University of Trento, Via Mesiano, 77, 38123 Trento, Italy; rida.jbr@unitn.it

**Keywords:** indoor air quality (IAQ), electrospinning technique, particulate matter (PM) removal, volatile organic compounds (VOCs), adsorption, biopolymers, waste upcycling

## Abstract

Air filters are crucial components of building ventilation systems. Compared to conventional air filter media like glass fibers and melt-blown fibers, electrospinning membranes are more efficient for capturing various pollutants due to the smaller pores present on the structure. In this paper, activated carbon filters were prepared with eco-friendly polylactic acid (PLA) and microcrystalline cellulose (MCC) using electrospinning to obtain a high-quality factor (QF) fibrous mat for aerosol particle matter (PM) filtration and volatile organic compounds (VOCs) adsorption. Several configurations of the final membranes were investigated and tested for fiber morphology and air filtration performance. Filtering efficiency and adsorption properties were evaluated in a real-scale room by measuring the particle penetration of the newly synthesized and commercial filters against neutralized aerosol particles (3% NaCl aqueous solution) and VOCs (methyl ethyl ketone). The calculated depolluting efficiencies were up to 98% in terms of PM and 55% for VOCs abatement, respectively. Our results indicate that the proposed hybrid membranes represent promising materials for highly efficient and sustainable air filters for home application systems.

## 1. Introduction

The built environment has a notable impact on the health and well-being of occupants, especially considering that the population spends 80 to 90% of their time indoors [1]. Indoor air quality (IAQ) refers to the air quality within and around buildings and structures, especially as it relates to the health and comfort of building occupants. Understanding and controlling common pollutants indoors can help mitigate health risks [2]. To enhance IAQ, several techniques can be employed, such as source control, ventilation, and air cleaning. Air cleaning is considered a valuable tool since it has a relatively low price and uses easy to implement technology [3]. For example, traditional borosilicate glass filters (HEPA) can be replaced with nanofibrous air filters that offer several advantages, including a high surface area, a low pressure drop, low thickness, and lightweight features [4]. Electrospinning is recognized as an efficient, economic, and versatile method [5] to prepare air filter media with nano and sub-micron fiber diameters [6,7]. The production of the fibers is obtained by a liquid droplet that is electrified to generate a jet during the electrohydrodynamic process of electrospinning, and, consequently, elongation of the solution is provided.

To enhance the sustainability of the filters, biodegradable and compostable biobased polymers such as cellulose or polylactic acid (PLA) have been demonstrated to be effective in the removal of particulate matter (PM) [8] or when doped with adsorbents—of volatile organic compound (VOCs) [4].

PLA is a biodegradable and recyclable aliphatic polyester produced from renewable feedstock. The monomer, lactic acid (LA), is obtained from bacterial fermentation of polysaccharides extracted from corn, sugarcane, potatoes, and other agricultural sources [9]. Due to its eco-friendly properties, PLA has been demonstrated to be a potential material to replace conventional petrochemical-based polymers for widespread applications. Due to its mechanical resistance, biocompatibility, and facility of processing characteristics [10], PLA has recently been used in various sectors [11] such as the medical/biomedical industry [12], packaging production [13], pharmaceuticals [14], and textile industries [15,16]. As a thermoplastic, PLA melts at 150–160 °C and can be processed using standard techniques such as extrusion, injection molding, film blowing, and thermoforming.

Despite its numerous advantages, PLA has some imperfections that limit its use in certain applications. The main limitations are: (1) poor toughness, as PLA is highly brittle with less than 10% elongation at break, and (2) a slow crystallization rate, which is crucial for controlling degradation. PLA degrades through the hydrolysis of backbone ester groups, and its degradation rate depends on factors such as PLA crystallinity, molecular weight, distribution, morphology, the water diffusion rate into the polymer, and stereoisomeric content. The degradation rate is often considered to be an important selection criterion for biomedical applications [17].

The addition of reinforcements is one strategy to improve PLA structural properties. These particles can be both, organic and inorganic, and the result of this combination is a matrix–fiber hybrid composite [18]. Enabling the formation of hierarchical structures on the PLA membrane surface is commonly achieved by incorporating inorganic substances, such as TiO_2_-sol or metal organic frameworks (MOFs), which results in the modification of their properties (improvement of surface roughness and wettability) [19]. The oil adsorption capacity of the neat PLA electrospun mats (7.75 g/g) increased by approximately 89% and 102% with the incorporation of molybdenum disulfide (MoS_2_) and tungsten disulfide (WS_2_), respectively [20].

On the other hand, among the organic fillers [21], microcrystalline cellulose (MCC) has been shown to improve the mechanical properties of the produced fibers [22]. MCC and nanocrystalline cellulose (NCC) are purified, partially depolymerized non-fibrous forms of cellulose. Both are produced by the purification and hydrolysis of cellulose. During the purification step, the lignin is destroyed and removed in raw materials using alkaline treatment. This step aims to remove the lignin, which can inhibit acid penetration during the next step (hydrolysis). The hydrolysis process aims to remove the amorphous parts of cellulose, giving short rod-like fibrils of high crystallinity [23]. Crystalline cellulose is mostly extracted from cellulose fibers using acid hydrolysis; however, it can be also isolated using enzymatic hydrolysis or extracted from different wastes [24,25]. Depending on the hydrolysis conditions, like type of acid and concentration, crystalline cellulose can be obtained in micro- to nano-diameter sizes. Nanocrystalline cellulose has a diameter smaller than 10 nm and lengths of 100–500 nm, while microcrystalline cellulose has lengths from 50 to 200 µm [26].

The use of cellulose micro- and nanocrystals as reinforcements in composites has attracted attention due to the increasing interest in developing new sustainable and environmentally friendly materials.

Rico et al. reported that MCC’s high crystallinity provides exceptional reinforcement due to its high modulus, with a longitudinal modulus of approximately 150 GPa, enhancing the mechanical properties of the resulting biocomposites [27]. The effect of mixing microcrystalline cellulose fluids (MCCFs) with PLA on the micropore morphology of PLA was assessed, revealing that an increase in MCCFs resulted in a reduction in the microporous surface of PLA fibers. This finding was attributed to the fast filling of MCCFs into micropore before solidification. In addition, the tensile strength of pure PLA fabric was 3.8 times lower than PLA/MCCF fabric [28]. Moreover, it was recorded that the elongation at break of PLA/MCCFs showed a nearly fourfold increase (48.84%) compared to that of pure PLA (13.19%). Additionally, incorporating waste-derived MCC into PLA/poly(butylene succinate) (PBS) composites results in sustainable biocomposites while reducing the overall cost of PLA [29]. This approach helps maintain the environmentally friendly nature of these materials while making them more economically viable. What is more, the cellulose crystals used for the reinforcement of PLA increase the rate of degradation, and the samples disintegrate very rapidly [30]. The valorization of cellulose, which is the most abundant renewable carbon-based material of our planet, offers an important alternative as an additive to polymeric matrix formulations [31].

Not only the structural characteristics but also the depolluting properties can be increased by the functionalization of electrospun filters with carbon-based materials such as activated carbon [32]. This possibility was previously explored, and the enhancement of VOCs removal was 60% higher than the reference material thanks to the addition of adsorbent material [33], while the bacterial filtration efficiency was up to 98% [11]. The sustainability of the final product can be increased also in this case thanks to the use of waste-derived materials [34].

The scope of this work involves the assessment of the possibility of the fabrication of electrospun biopolymeric filters and their combination with powdered activated carbon to obtain a hybrid filter. The novelty of this work resides in the use of a sustainable source of cellulose that comes from textile waste streams and the possibility of integration into PLA electrospun solutions to enhance its properties. In addition, to the best of the authors’ knowledge, there are no studies about multipurpose filters fabricated with electrospinning and prepared with PLA microcellulose and activated carbon from waste materials that are able to abate not only PM but also VOCs. New formulations have been designed and characterized in terms of morphology, thermal stability, and chemical composition. The particulate matter (PM) filtration efficiency of the electrospun filters and the VOCs adsorption efficiency of the hybrid filter are studied in view of a possible application of this innovative filter as an odor reduction and PM filtration unit.

## 2. Results and Discussion

### 2.1. Morphological Investigations

The efficiency of filtering materials depends on their fiber structure, morphology, and porosity [7,35]. FESEM analysis was performed to analyze the morphology of traditional (commercial) and PLA-based filters. The filters were prepared based on previously optimized parameters [4], and the influence of the substitution of PLA pellets with MCC was investigated. Fibers were successfully produced in all samples except for (D) 13% PLA and (D) 14% PLA, as shown in Figure S4 in the Supplementary Materials. The lack of fiber formation and the presence of beads from the 13% PLA and 14% PLA solutions suggest an unstable jet. As reported in the literature, beads form when the surface tension and viscous forces form an unstable jet, and the liquid properties approach those leading to capillary break-up [36].

**Figure 1:**
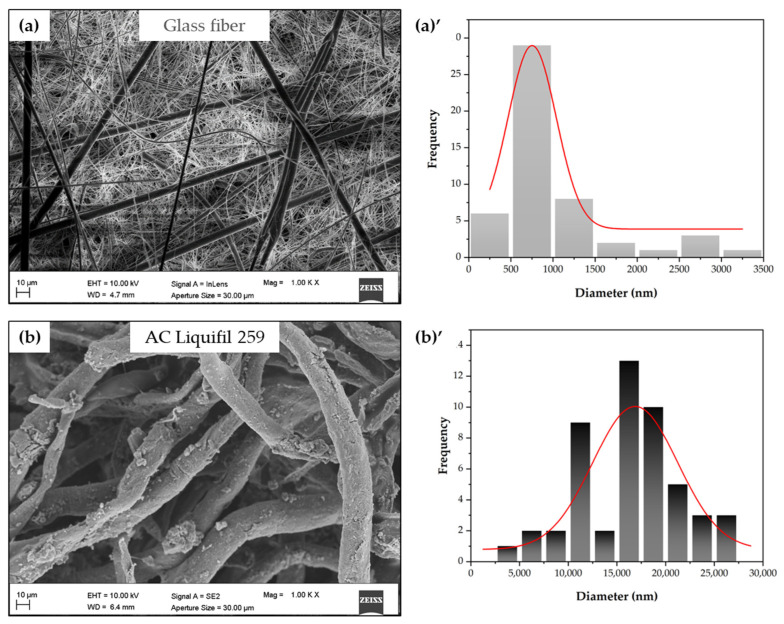
Morphological analysis of commercial filters: FESEM images of glass fiber (**a**), and AC Liquifil 259 (**b**), and diameter fiber distributions ((**a**)’, (**b**)’) of commercial filters.

**Figure 2:**
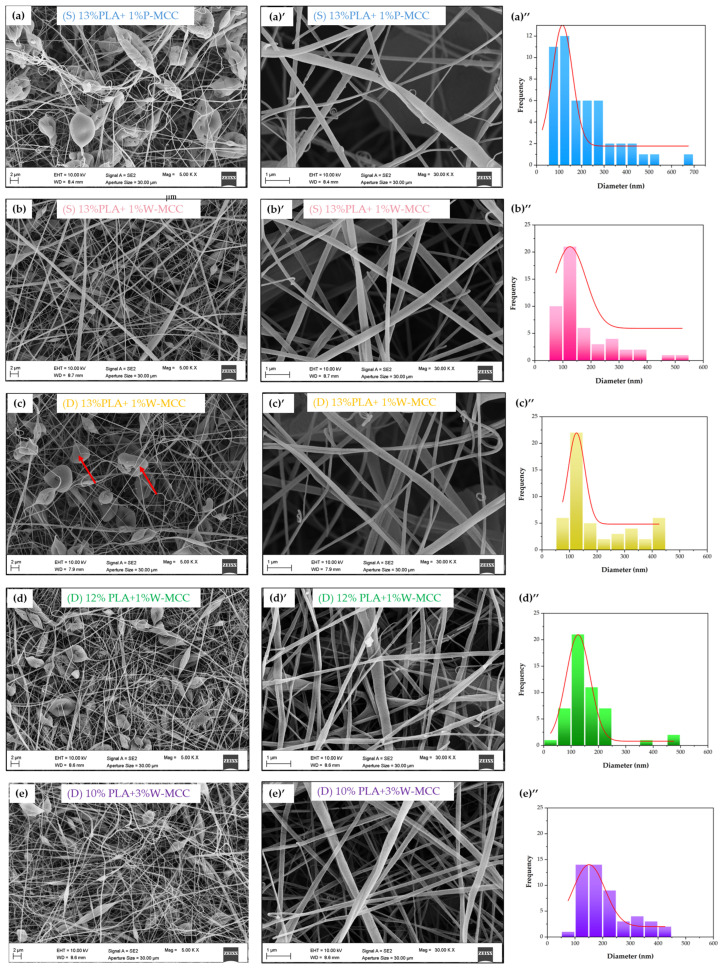
Morphological analysis of fabricated filters: FESEM images ((**a**)–(**e**), (**a**)’–(**e**)’) and diameter fiber distributions ((**a**)’’–(**e**)’’) of electrospun PLA+W-MCC fibers.

**Figure 3:**
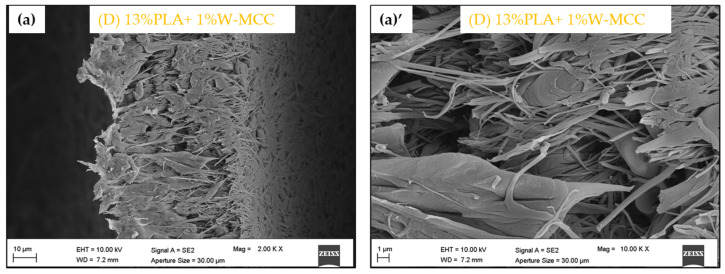
FESEM images ((**a**), (**a**)’) of the cross section of (D) 13% PLA+1% W-MCC.

The images of commercial filters are shown in Figure 1. The images of PLA-MCC and PLA fibers are presented in Figure 2. Figure 3 displays the cross section of the (D) 13% PLA+1% W-MCC filter. Since it is not possible to detect the MCC particles, it suggests that they are arranged inside and along the fibers. The effect of its addition is an increase in the diameter of the fibers, not the average one as detected in [37] but the maximum diameter, as shown in Table 1.

**Figure 4:**
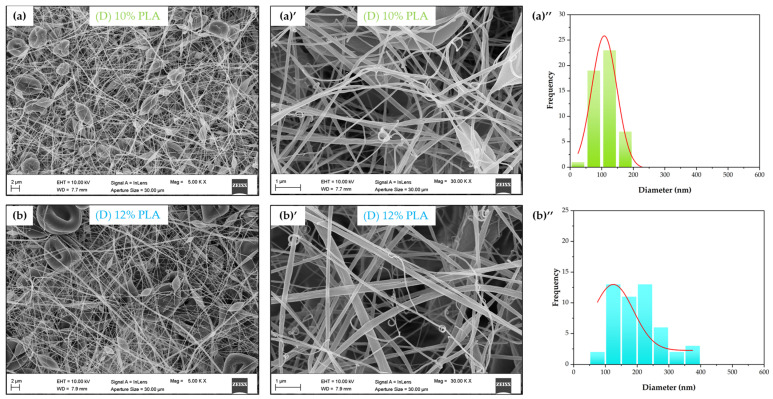
Morphological analysis of fabricated filters: FESEM images ((**a**),(**b**), (**a**)’,(**b**)’) and diameter fiber distributions ((**a**)’’,(**b**)’’) of electrospun PLA fibers.

The commercial AC Liquifil 259 (Figure 1b) and glass fiber (Figure 1a) filters have the highest fiber diameter, while the 10% double-layer PLA ((D) 10% PLA) (Figure 4a) filter has the lowest average diameter. The higher the total polymer concentration is, the higher the fiber diameter.

The glass fiber filtering medium consists of randomly oriented fibers with a very wide range of diameters, ranging from 356 nm to 3.2 µm (Figure 1a).

The fibers obtained from electrospinning of PLA are broken or twisted, and this series may indicate nonoptimal electrospinning conditions (Figure 2). However, it is notable that the addition of MCC improved the morphology of the fibers. For the PLA/P-MCC filters, the fiber diameter ranges from a minimum of 66 nm to a maximum of 673 nm, with an average diameter of 205 nm. All samples exhibit the presence of beads, which plays a role in the efficiency of the filter and the pressure drop given that the space between the fibers is reduced by these features. A similar bead-like structure was obtained by Heidari and coauthors when MCC and PVA were mixed at a 45:50 ratio [38]. The different shapes of beads between the filters containing P-MCC could be due to the different hydrolysis conditions and type of starting source, which affect the particle morphology, crystallinity, and molecular weight of MCC and, hence, influence the spinnability and morphology of the produced fibers [39]. On the other hand, the addition of waste-derived cellulose (W-MCC) at 1% concentration results in a better structure, with a low number of beads (ellipse shaped) and no twisted fibers, suggesting that higher viscosity is achieved. The formation of beads can be caused by a viscosity of the solution that is relatively low [40] compared to the spinning systems, so the balance among the viscoelasticity, surface tension, and electrostatic repulsion of the jet becomes disrupted, affecting the formation and the stability of the Taylor cone [41]. Apart from the viscosity (concentration) of the polymers, the applied electric current during the electrospinning process also affects fiber morphology due to fact that the shape of the Taylor cone is affected by the applied voltage and the distance between the needle and collector [42]. The nanofiber diameter distribution graphs show that with the addition of W-MCC, the diameter distribution of (D) 12% PLA+1% W-MCC and (D) 10% PLA+3% W-MCC are shifted to the larger side with respect to (D) 12% PLA and (D) 10% PLA.

In the case of double-layer filters, the transition between the two layers is not detectable, which indicates optimum adhesion. This result was achieved using a longer electrospinning process for the preparation of fibers. This is evident in Figure 3, where the cross section is analyzed. In this case, it is evident that the addition of MCC caused a higher average diameter of the fiber [43].

The presence and the influence of microcellulose from waste addition was also investigated using FT-IR and TG. For this analysis, the most representative samples were studied, namely, (D) 10% PLA+3% W-MCC and (D) 10% PLA.

**Figure 5:**
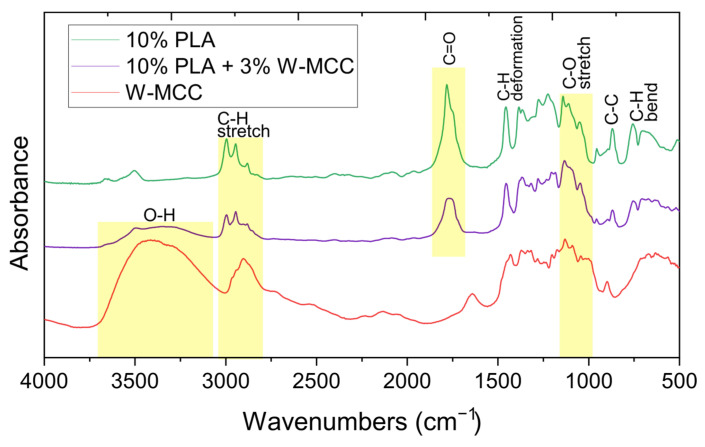
FT-IR spectra of 10% PLA, 10% PLA+3% W-MCC, and W-MCC.

Figure 5 shows the results from FT-IR analysis. In the PLA sample, the peak observed at approximately 1750 cm^−1^, corresponding to C=O stretching, appears asymmetric and is likely split into two components (up to four split components, likely due to carbonyl–carbonyl coupling or dipole interactions rather than hydrogen bonding [44]). In the (D) 10% PLA+3% W-MCC sample, the higher wavenumber component of this split peak exhibits a reduction in intensity. This change may result from interactions between PLA and MCC, which can influence the carbonyl groups. The O–H stretching vibration (~3350 cm^−1^) is present but significantly weaker in the (D) 10% PLA+3% W-MCC sample compared to W-MCC, and it is absent in PLA. The reduced intensity in the PLA + MCC system suggests hydrogen bonding between PLA and MCC, which can restrict the vibrational freedom of hydroxyl groups. Additionally, a small shift in the C–O stretching peak of cellulose is observed, shifting from ~1050 cm^−1^ to ~1045 cm^−1^ in the (D) 10% PLA+3% W-MCC sample. This shift may indicate interactions of the cellulose with PLA, possibly related to hydrogen bonding or conformational modifications in the polymer matrix.

**Figure 6:**
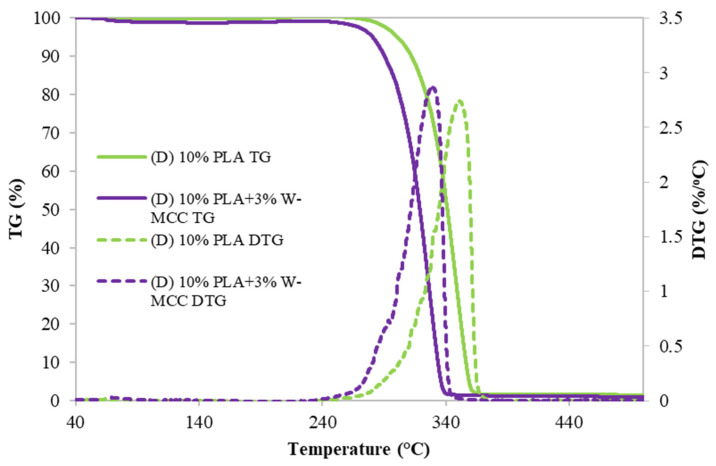
TG and DTG profiles of (D) 10% PLA and (D) 10% PLA+3% W-MCC.

In order to investigate the thermal properties of the PLA and PLA/MCC filters, TG and DTG were performed, and results are presented in Figure 6.

Looking at the DTG curve of PLA ((D)10%PLA), the peak observed around 350 °C implies the typical decomposition of PLA in one-stage weight loss [45] based on the dehydration reactions and the generation of volatile products through chain scission and decomposition.

When MCC (sample (D) 10% PLA+3% W-MCC) is present in the filter, the DTG peak shifted to a lower temperature, around 330 °C, which is around 10 °C lower than pure PLA [46]. The shift is due to the presence of MCC that has a lower decomposition temperature than PLA. Its presence modifies the overall behavior of the filter, and the location of these two peaks was consistent with those of MCC and PLA [47]. Same behavior was detected in [48], where PLA and cellulose composites were studied.

### 2.2. PM Filtration

**Figure 7:**
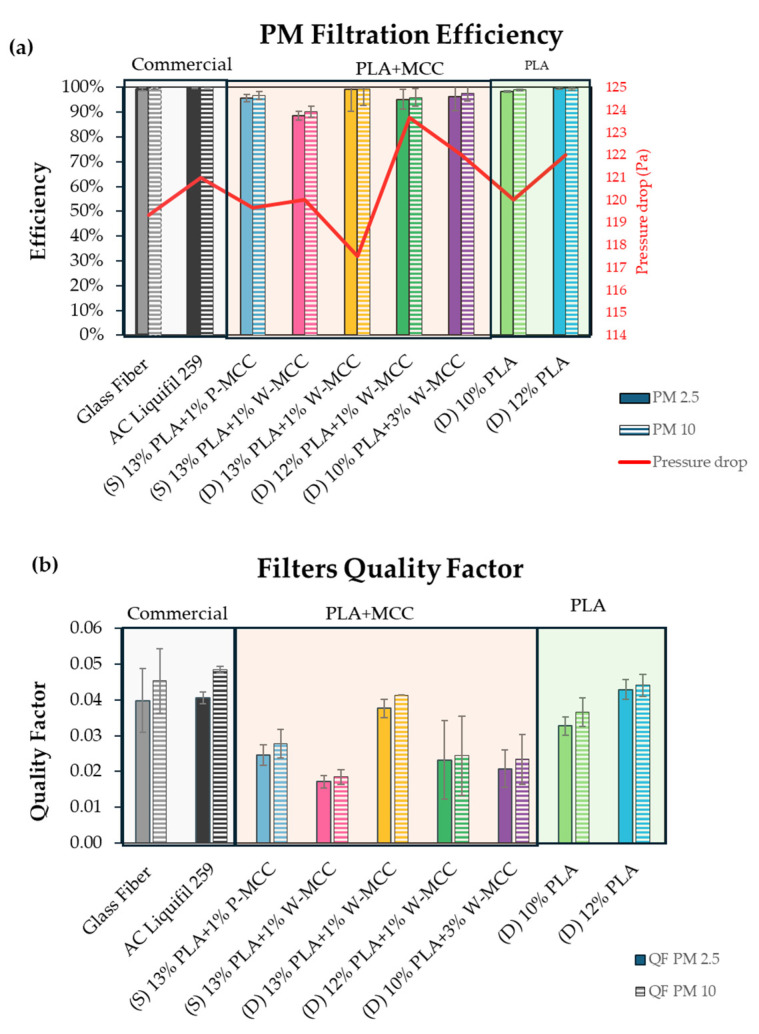
(**a**) Filtration efficiency results and (**b**) quality factors.

The filtration efficiency test results are presented in Figure 7.

Two particulate matter sizes have been monitored: PM2.5 and PM10. All electrospun filters exhibit very high filtration efficiency, with values over 90%, which according to the EN779-ISO16890 [49] classification system, classify them as class F8 or F9. In contrast, commercial glass fiber filters show the highest removal efficiency, with values over 99% for PM10 and PM2.5. The pressure drop ranged from 118 to 124 Pa. Due to the lack of fiber formation from the 13% PLA and 14% PLA solutions, these samples were not tested for PM filtration.

The (S) 13% PLA+1% P-MCC sample demonstrated excellent filtration efficiency, with a decrease in efficiency of 4% (for PM2.5) and 3% (for PM10) compared to the commercial glass fiber. The addition of 1% of waste-derived MCC decreased the filtration efficiency by 7% (PM2.5) and 6% (PM10) compared to the filter with the addition of the same amount of P-MCC. To increase the structural properties of the filters and address the difficulty in separating 10 mL solution filters from the collector due to their thin and brittle nature, double-layer filters (D) were produced using twice the amount of solution (20 mL). This step did not significantly affect the drop pressure, which ranged from 118 to 124 Pa for double-layer filters. On the other hand, the effect of a double-layer filter, namely, (D) 13% PLA+1% W-MCC, on PM filtration was relevant. In fact, an excellent removal efficiency of 95% for PM2.5 and 96% for PM10 was detected. This led to increases in the filtration efficiency of 6% for PM2.5 and PM10 compared with the same single-layer filter. The next two filters, (D) 12% PLA+1% W-MCC and (D) 10% PLA+3% W-MCC, concern the attempt of the substitution of PLA with MCC. If the final percentage of PLA/MCC is considered, the obtained filters consisted of 95% PLA and 5% W-MCC and 80% PLA and 20% W-MCC, respectively, as shown in Table 2. The filtration efficiency of (D) 12% PLA and (D) 10% PLA were compared to (D) 12% PLA+1% W-MCC and (D) 10% PLA+3% W-MCC to understand the effect of MCC addition in the filter.

The results showed that the removal efficiency was comparable to the filter without W-MCC in both cases, with a slight decrease in the filtration efficiency (from 8% to 5%).

The AC Liquifil 259 filters showed very high removal efficiency. The high efficiency is not attributable to the activated carbon, which does not have PM filtration activity, but rather to the polymeric layer that the activated carbon is based on.

The pressure drop values also are shown in Figure 7a, and this is a crucial parameter for air filters that is strictly related to efficiency and directly impacts energy consumption [50]. The lowest value of the pressure drop is detected for (D) 13% PLA+1% W-MCC, which shows an average diameter of fiber of 196 nm. In contrast, the highest value is noted for (D) 12% PLA+1% W-MCC, which has a lower average diameter for the fibers (159 nm). On the other hand, the effect of fiber diameter on pressure drops is more evident for pure PLA filters where no particles can affect the behavior. In fact, changes in porosity can occur due to bead formation. In pure PLA filters, 10% PLA had average diameter of 111 nm and a pressure drop 120 Pa. However, for 12% PLA, the average diameter was 201 nm, and the pressure drop was a bit higher at 122 Pa. Thus, the lower the diameter of the fiber is, the lower the pressure drop. This is probably due to the increase in air penetration and an increase in the effective surface area and surface roughness. The same results were also discussed in [51].

The QFs are also shown in Figure 7b. The highest QF is related to the commercial AC Liquifil 259 filter. As mentioned before, the high particulate matter removal performance of this filter is attributable to its polymeric latex base network. The quality factor of the (D) 12% PLA filter is comparable to the glass fiber B filter, followed by the second (D) 10% PLA with QFs of 0.037 Pa^−1^ and 0.039 Pa^−1^ for PM2.5 and PM10, respectively.

In case of the tested filters, the particles are captured using various mechanisms, and the diffusion model can describe the electrospun filters [52]. Since the fiber diameters of PLA fall from 130 to 240 nm, the Knudsen number falls within the transition and slip flows [53,54].

**Figure 8:**
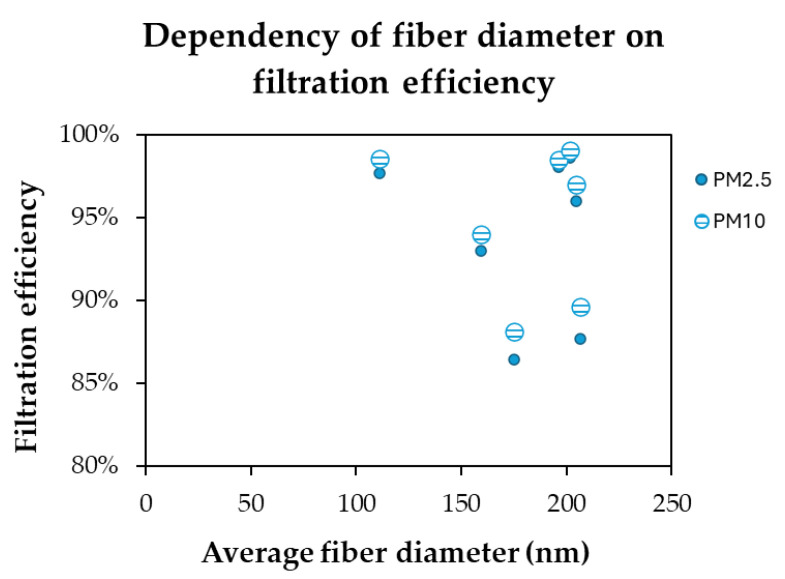
Relationship between the fiber diameter and filtration efficiency of PLA-based filters.

As shown in Figure 8, the relation between the fiber diameter and the filtration efficiency was detected. The tested PLA filters, when compared to the commercial one, show also a relevant difference in the fiber diameter but not in the quality factor. The main motivation is the relatively constant fiber diameter distribution within the same type of filter. In fact, the variation in fiber diameters creates the possibility of competition between the fibers for particle capture [55]. For example, microfibers are less exposed to the air streams than nanofibers, so less particles can be intercepted by the fibers. From this point, the nanofiber behavior depends not only on the morphological properties, but also thickness. In general, thinner fibers result in higher efficiency, but also a lower permeability/pressure drop, while thicker fibers have lower efficiency and lower permeability [55]. This can overall be enhanced for small PM particles, such as PM0.3 [56].

**Figure 9:**
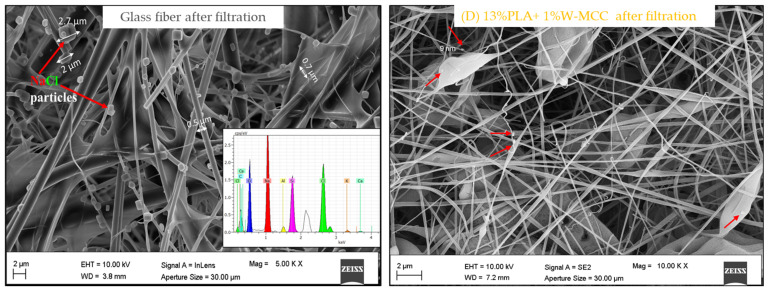
FESEM images of glass fiber and 13% PLA+1% W-MCC after the filtration test. Filtered particles are indicated with red arrows.

The presence of NaCl particles was demonstrated to be intercepted by the fibers of the filter. This is shown in Figure 9, where the FESEM+EDS analysis is reported.

The elemental composition of the observed particles is provided, and the geometrical diameter is also reported. The EDS confirms that the trapped particles are mainly composed of sodium and chloride. These particles result from the humidifier that dispersed tiny droplets of the NaCl solution in the air (aerosolization). As water evaporates, the NaCl particles were left suspended in the nebulized air and then intercepted and trapped by the filtering media [53].

### 2.3. VOCs Adsorption

**Figure 10:**
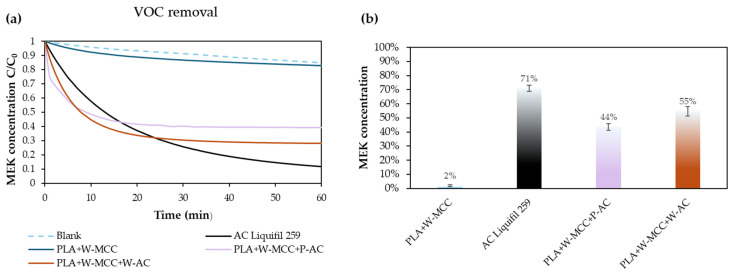
(**a**) VOCs adsorption profiles and (**b**) comparison of VOCs removal.

The results of the VOCs adsorption are reported in Figure 10, where the C/C_0_ ratio evolution during the test is shown. Many authors suggest that the potential VOCs removal capacity involves π–π interactions, hydrophobic effects, and van der Waals interactions [43]. When no nanoparticles, such as metal oxide, are inserted in the formulation [57], the nanofiber exhibited considerable selectivity and the best thermal desorption efficiency [58]. For this reason, the filters were doped with high adsorbent material such as activated carbon that permits lowering the selectivity of VOCs.

The PLA+W-MCC filter without the addition of activated carbon does not show VOCs adsorption properties, and the monitoring line (blue line) after 60 min of the test starts to overlap the blank line (dashed line). This means that the adsorption process is not occurring, and only the natural decay of MEK into the test box was monitored. The decrease follows an asymptotic trend for all commercial and hybrid filters (PLA+W-MCC+P-AC and PLA+W-MCC+W-AC). The MEK concentration shows a sharp decrease in the first 20 min and then an almost constant plateau in the next 40 min of the test. This indicates that the adsorption sites are saturated after 20 min of the test, and no further adsorption can take place. In the case of the PLA/MCC filters, decreases of 44% and 55% was detected when pure or waste cellulose was used, respectively. If the activated carbon was not added to the filters, the adsorbent capacity would be missing, as [59] found, where the capacity of adsorbing VOCs up to 33% was reached thanks to the addition of active carbon, which was sprayed on the surface of the filters. The commercial AC filter (AC Liquifil 259) does not present the flat trend after 20 min, but the adsorption carries on for the length of the test. However, as reported in Table 3, the amount of active carbon present in this commercial filter is indeed much higher than the active carbon content on PLA/MCC filters; hence, the adsorption sites are not easily saturated.

**Figure 11:**
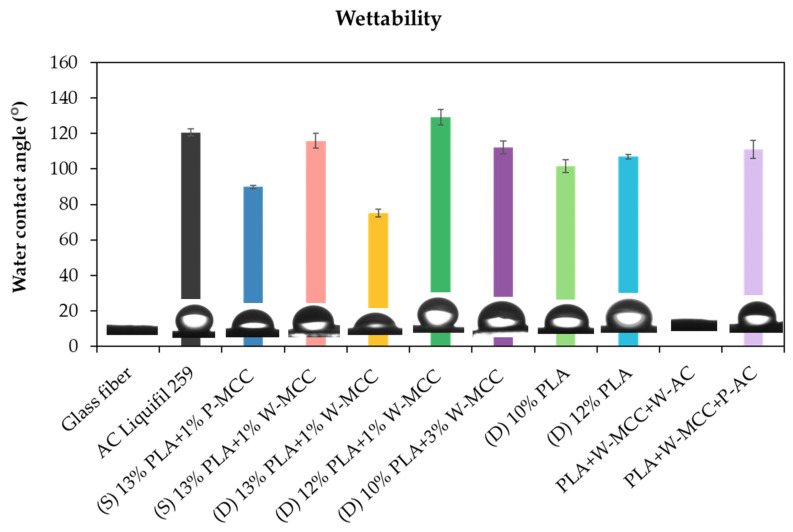
Comparison of the contact angle values for PLA and PL/MCC fibers.

The wettability of all electrospun samples was investigated by measuring the contact angle (CA) of droplets of deionized (DI) water on their surface, and the results are presented in Figure 11. For the glass fiber and PLA+W-MCC+W-AC samples, the values were not detected. The surface exhibited complete wetting behavior, and the contact angle was not measurable due to its complete hydrophilicity. Additionally, it can be noticed that an increase in the total polymer concentration to 14% in the double-layered samples caused a decrease in the water contact angle. This indicates the hydrophilic nature (CA < 90°) of the following samples: (D) 13% PLA+1% W-MCC and (D) 14% PLA. The values are 75.1 ± 2.3 and 58.5 ± 4.7, respectively. Moreover, it has been reported that higher water absorption (lower contact angle) depends also on the fiber content. In fact, the lowest value is indicated for (D) 14% PLA (Table S2 in Supplementary Materials), where the fibers were not produced [60]. However, this phenomenon was not observed for (D) 13% PLA.

It is expected that the addition of MCC into PLA network would increase hydrophilicity of PLA samples due to the hydrophilic nature of MCC [61]. However, the opposite phenomenon was noticed. In all the samples, the addition of MCC caused a slight increase of the water contact angle compared to the samples without MCC, excluding sample (D) 13% PLA+1% W-MCC.

## 3. Materials and Methods

### 3.1. Materials

Polylactic acid (PLA) from Total Corbion Luminy^®^ LX930 with a density of 1.24 g/cm^3^, glass transition temperature (Tg) of 60 °C, and melting point (Tm) of 130 °C was used, and acetone (Sigma Aldrich, St. Louis, MI, USA, ≥99.5%) was used as a solvent. Two different types of microcrystalline cellulose (MCC) were used and compared: (i) a pure grade Merc Millipore (MCC) for gas chromatography (P-MCC) and (ii) waste-derived MCC extracted from textiles, labelled as W-MCC. Commercial Carbosorb PCF (COMELT S.p.A) (labelled as P-AC) and biomass waste-derived (W-AC) activated carbons were used to functionalize the electrospun filters, with specific surfaces of 1200 m^2^/g and 1597 m^2^/g, respectively. The VOCs adsorption efficiency was 3046 mg/g for P-AC and 3210 mg/g for W-AC, as described and tested in a previous study [34].

The electrospun filters were also compared to two different commercial filters: a nonwoven mat of borosilicate microglass fiber used as a normal HEPA-grade filter (labelled as glass fiber) and AC Liquifil 259 manufactured by Purification Products Limited, Leeds, UK. AC Liquifil 259 is an air-laid web material consisting of powdered activated carbon and cellulose wood pulp fibers bound onto a cotton backing with a modified acrylonitrile copolymer latex. AC Liquifil 259 is used for heavy duty or intense filtration applications.

W-MCC was extracted from textile wastes using controlled acid hydrolysis, and the recovery procedure is briefly described. Briefly, 5 g of cotton waste was chemically treated by mixing with 200 mL of 20% H_2_SO_4_ for 5 h at 80 °C. After chemical treatment, the reaction mass was centrifuged (10 min, at 3500 rpm) to remove the supernatant. The sediment was neutralized under magnetic stirring using a sodium hydroxide solution. The mass was then filled up to its original volume, and a second centrifugation was performed. The samples were frozen at approximately −27 °C and lyophilized. The obtained cellulose powder was then sieved (Retsch vibration screening machine, RETSCH GmbH, Haan, Germany) to obtain fraction of particles < 100 µm.

### 3.2. Electrospun Filter Preparation

The preparation process of the electrospun filters involves two steps. The first step involves the production of the membrane. This is followed by functionalization using activated carbon.

PLA/MCC fibers were fabricated by using an electrospinning technique. A horizontal collector with aluminum foil was used, and the ambient conditions during the electrospinning process, such as temperature and relative humidity, were recorded. The solvent (acetone) was added to the polymer during the preparation process, and MCC was mixed with PLA pellets in various concentrations, ranging from 1% to 3% *w*/*w* total concentration in the solution (from 5% to 20% compared to PLA weight), to obtain the final total polymer concentration of 13% *w*/*w* or 14% *w*/*w*. To evaluate the effect of the MCC addition, 10%, 12%, 13%, and 14% PLA solutions were also prepared. The mixtures were then mixed to obtain uniform solutions that were then electrospun using an electrospinning device (Spinbox, Fludinatek, Valencia, Spain). The polymer solutions were poured into a polypropylene syringe with 20 mL capacity connected to a stainless-steel needle (22 gauge). A high positive voltage (20 kV) was applied between the needle and the flat collector that was placed at the distance of 10 cm from the needle, and the syringe pump rate was set at 0.07 mL/min based on previous studies and literature evidence [53]. The electrospinning process was stopped after 2 h 40 min (target volume of the electrospun solution 10 mL) or 5 h and 20 min of spinning (target volume of the electrospun solution 20 mL) of the polymeric solution and the samples were labelled as single- (S) or (D) double-layer filters, respectively. During electrospinning, T and RH were recorded at 20–26 °C and 40–56%, respectively. At least three round shaped samples were cut with a diameter of 6 cm from electrospun mats and stored for the analysis.

The last step involves hybrid filter preparation. Activated carbon was sprayed on the electrospun surface using the airbrush coating method [62,63], obtaining the adsorptive layer. The reason why activated carbon was not mixed with the polymer solution before the electrospinning is to avoid blocking the pores present in the activated carbon particles by the layer of the polymer [52]. The activated carbon was first crushed until passing at 63 µm to further decrease the dimension of the grains and break the thicker bulks; this was necessary to avoid the clogging of the airbrush during the spraying operation. In order to upcycle the polymer waste using electrospinning, samples fabricated from waste cellulose were selected (PLA-W:MCC). The filter was first weighed, and then the crashed activated carbon was dispersed in the ethanol and sprayed with an airbrush on the filter surface. Ethanol was chosen as a solvent because it does not dissolve in PLA, and it evaporates at a faster rate than water. After the complete evaporation of the solvent, the filter was weighed again to determine the quantity of activated carbon deposited to the polymeric base. As described in Section 3.1, three types of activated carbons were used as adsorbents. These included commercially available P-AC and organic waste W-AC, and the commercial polymeric activated carbon filter (AC Liquifil 259) was used as a reference standard for the adsorption efficiency.

Lists of the tested filters in the PM and VOCs removal tests are shown in Table 2 and Table 3, respectively. Additionally, the activated carbon content present in the filters and reported as a percentage of weight is shown in Table 3.

### 3.3. Electrospun Filter Properties

A field emission scanning electron microscope (FESEM—Zeiss Supra 40, Oberkochen, Germany) was used to study the morphology of the electrospun and commercial filters. The average diameters and the diameter distribution were calculated using ImageJ (version 1.54g) based on at least 50 random diameter measurements from the corresponding FESEM images, and the distribution, minimum, maximum, average, and mean diameters values are reported. FESEM and elemental composition (FESEM+EDS) analysis was also provided to support the results obtained from filtration tests. In fact, the surfaces of the filters were analyzed both before and after the exposure to PM test, so the particles trapped by the filtering media were analyzed [52].

The chemical characterization of the filters was carried out using Fourier transform infrared spectroscopy (FT-IR). The IR spectra were recorded using a Bruker Invenio-R spectrometer (Bruker, Karlsruhe, Germany) equipped with either a Ge ATR-crystal or by transmittance. All spectra were measured based on the average of 128 scans.

In order to explore the thermal behavior of composites at a higher temperature to qualify composites for thermally sensitive applications, the mass loss was determined using thermo gravimetric analysis (TG) curves and mass loss derivatives (DTG) of filters. Thermo gravimetric analysis (TGA) was performed using TG/DTA6200, Exstar 6000, and SII (Hitachi High-Technologies Corporation, Tokyo, Japan) under a 300 mL/min nitrogen atmosphere using a heating rate of 10 °C/min.

Water contact angles were measured using a custom-built setup equipped with a Thorlabs C1280G12C camera (Thorlabs, Newton, NJ, USA).

Raman spectroscopy was applied to characterize W-MCC and P-MCC. The spectra were collected using an Xplora Plus Raman microscope (Horiba, Tokyo, Japan) with a 785 nm laser as the excitation source to minimize fluorescence from microcellulose. Here, 600 grating and a 100× objective were used for the measurements.

#### 3.3.1. PM Removal

**Figure 12:**
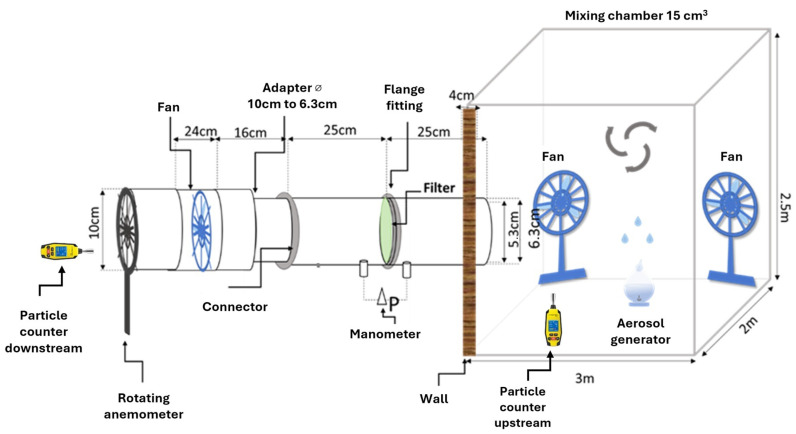
Schematic diagram of the testing room (figure not to scale).

The PM removal efficiency of the electrospun filters was evaluated in a 15 m^3^ room with two rotating fans as shown schematically on Figure 12. The filter is placed inside a sample holder with a 6.3 cm external diameter made of 3D printed PLA inside a PVC pipe. The pipe has a pumping system that permits a constant flux of (200 L/min). To avoid measurement disturbances, a series of small tubes (0.5 cm diameter) are inserted in proximity of the filter to ensure regular flow across the entire section. The PM was generated inside the test room thanks to a humidifier (Medisana, Neuss Germany) with a 3% NaCl w/V aqueous solution. The humidifier disperses tiny droplets of the solution in the air (aerosolization). As water evaporates, NaCl particles were left suspended, generating airborne PM. Two Trotect BQ21 particle counters are used to measure the PM concentration inside and outside the test room, immediately after the pipe. These particle counters use light-scattering measuring technology to detect and quantify PM. The device has two channels, which are designed to measure particles with aerodynamic sizes of 2.5 µm and 10 µm, respectively, as internationally recognized categories of fine particulate matter (PM2.5 and PM10, respectively). The test started when the humidifier was turned on inside the room. After 15 seconds, the humidifier was turned off to prevent exceeding the maximum concentration detectable by the instrument, which is equal to 2000 μg/m^3^. The test was carried out for a total of 5 min. The calibration tests results are presented in Figure S3.

For this system, the percentage filtration efficiency (E) was calculated according to the following equation:

$$E\;(\%)=\left(1-\frac{C_{downstream}}{C_{upstream}}\right)\cdot 100$$

where C_downstream_ and C_upstream_ are the PM measurements (µm/m^3^) inside the room before the filter and after the filter, respectively.

The pressure drop generated by the filter was measured with a Trotec TA400 manometer (Trotec, Marchtrenk, Austria), and the quality factor (QF) was calculated using the following equation:

$$QF\;({Pa}^{-1})=\frac{-ln[(1-E)]}{\Delta P}$$

where E and ΔP represent the filtration efficiency and pressure drop, respectively.

#### 3.3.2. VOCs Adsorption

The filters were placed on the extremity of a PVC tube with 6 cm diameter just after a small turbine; the turbine creates negative pressure and forces the air through the filter. The tube was then placed inside a sealed box (17 L) where 1 µL of VOCs tracer (methyl ethyl ketone—MEK) was injected thanks to a micro-syringe to ensure an initial concentration of 16 ppm (±1 ppm). after evaporation A fan was placed at the bottom of the reactor to guarantee continuous air mixing. To monitor changes in the VOCs concentration over time, a photoionization detector (PID) (Aeroqual, Auckland, New Zealand, Series 900, 0–30 ppm range) was used. The test was carried out for 60 min. Before each test, a blank test (without specimen) was performed to assess the natural decay of the VOCs concentration.

The percentage efficiency of VOCs removal was calculated according to the following equation:

$$Efficiency(\%)=\left(\frac{C_{0}-C_{f}}{C_{0}}\right)\cdot 100$$

where C_0_ is the concentration at time zero inside the box after the injection of the VOCs, and C_f_ is the concentration at the final time of the test inside the box. In cases involving monitoring over time, instead of C_f_, the concentration C_i_ (at time i) was considered.

## 4. Conclusions

Fabrication of polymer-based filters with the electrospinning technique has yielded optimum porous-based materials that are able to improve indoor air quality. The novelty of this study is twofold: (i) the use of all renewable, recovered, and biodegradable materials for manufacture, providing complete circularity from a materials perspective, and (ii) the use of a combination of polymeric fibers and adsorbent particles to extend the purifying properties to two ubiquitous indoor pollutants, PM and VOCs.

The study of the effect of the addition of MCC, both from commercial sources and recovered from waste, on the electrospinning process and on the final filter performance, both in terms of filtration of PM and mechanical properties, has been conducted. The waste-derived MCC was able to improve the morphology and consequently the mechanical performance of the filter. VOCs removal was guaranteed by means of adsorption thanks to the addition of waste-derived activated carbon deposited on the surface. To assess the possible application as an air depolluting material, the obtained filters were compared to commercial glass fiber and activated carbon filters. PM removal was studied, and all the tested filters exhibit a very high PM removal efficiency of up to 90%. The quality factors for the particles of the filter made from PLA and waste MCC are quite similar to those of commercial glass fiber and activated carbon filters. The addition of a small amount of waste-derived activated carbon by air spraying the fibers offers adsorbent behavior against VOCs (MEK) similar to that of commercial activated carbon filters. However, the saturation capacity cannot be compared given the noncomparable amounts of activated carbon involved.

The morphology of the fibers was investigated, revealing that cellulose addition increased the maximum fiber diameter. The presence of waste MCC in small quantities causes the diameter of only a small fraction of the fibers to increase. This does not penalize the filter’s properties toward PM, but represents a strategy to improve the filter’s workability, in other words, its mechanical properties. In the light of the very interesting PM and VOCs purifying properties, the path is open for future investigations aimed at characterizing the mechanical performance of filters through the realization of dedicated test specimens.

Thanks to this study, it has been possible to demonstrate the effectiveness of obtaining environmentally friendly materials (biopolymer) using the electrospinning method. In conclusion, electrospinning is a versatile and effective technique for producing composite materials with tailored purifying properties against specific pollutants. Particularly interesting is the possibility of valorizing waste materials without penalizing filter performance in the slightest.

## Figures and Tables

**Table 1:** Analysis of fiber diameter: minimum, maximum, average, and mean values.

Sample	Fiber Diameter			
	Min nm	Max nm	Average nm	Mean nm
Glass Fiber	356	3.2 × 10^3^	1.0 × 10^3^ ± 636	753 ± 71
AC Liquifil 259	4650	26.2 × 10^3^	1.6 × 10^3^ ± 5183	1.7 × 10^3^ ± 1114
(S) 13% PLA+1% P-MCC	66	673	205 ± 133	115 ± 10
(S) 13% PLA+1% W-MCC	75	521	175 ± 102	125 ± 30
(D) 13% PLA+1% W-MCC	84	432	196 ± 115	126 ± 10
(D) 12% PLA+1% W-MCC	49	489	159 ± 86	126 ± 7
(D) 10% PLA+3% W-MCC	98	425	207 ± 88	151 ± 17
(D) 10% PLA	42	173	111 ± 33	108 ± 2
(D) 12% PLA	89	378	201 ± 74	125 ± 46
(D) 13% PLA	No fibers			
(D) 14% PLA	No fibers			

**Table 2:** List of the prepared samples for the PM removal test.

Sample	Polymer Concentration	PLA	P-MCC	W-MCC	PLA:MCC Ratio	Solvent	Layers
Glass Fiber	Commercial						
AC Liquifil 259	Commercial						
(S) 13% PLA+1% P-MCC	14%	12.9 g	0.7 g	-	95:5	86.4 g	1
(S) 13% PLA+1% W-MCC	14%	12.9 g	-	0.7 g	95:5	86.4 g	1
(D) 13% PLA+1% W-MCC	14%	12.9 g	-	0.7 g	95:5	86.4 g	2
(D) 12% PLA+1% W-MCC	13%	12.4 g	-	0.7 g	95:5	87.0 g	2
(D) 10% PLA+3% W-MCC	13%	10.4 g	-	2.6 g	80:20	87.0 g	2
(D) 10% PLA	10%	10.4 g	-	-	100:0	89.6 g	2
(D) 12% PLA	12%	12.4 g	-	-	100:0	87.7 g	2
(D) 13% PLA	13%	13 g	-	-	100:0	87.0 g	2
(D) 14% PLA	14%	14 g	-	-	100:0	86.0 g	2

**Table 3:** List of the filters assessed in the VOCs removal test.

Sample	Filter Weight (mg)	Type of AC	AC (mg)	AC Content (%)
AC Liquifil 259	2134	Commercial	1067	* 50%
PLA+W-MCC	84.9	-	-	0%
PLA+W-MCC+P-AC	107.6	Commercial	22.7	21%
PLA+W-MCC+W-AC	93.7	Organic waste derived	13.9	15%

* Declared in data sheet.

## Data Availability

The original contributions presented in this study are included in the article/Supplementary Material. Further inquiries can be directed to the corresponding authors.

## Supplementary Materials

The following supporting information can be downloaded at: https://www.mdpi.com/article/10.3390/molecules30061214/s1, Figure S1. Raman spectra and images of P-MCC and W-MCC. Figure S2. Calibration of PM filtration system (blank test 1). Figure S3. Calibration of PM filtration system (blank test 2). Figure S4. Morphological analysis of (D) 13%PLA and (D) 14%PLA samples. Figure S5. Comparison of the contact angle values for PLA fibers. Table S1. Results from calibration test. Table S2. Water contact angle results for (D) 13% PLA and (D) 14% PLA.
