# Supplementary material for: Electrospun Fibers from Biobased and Recycled Materials for Indoor Air Quality Enhancement
Source: Molecules. 2025 Mar 8;30(6):1214. doi: 10.3390/molecules30061214 (PMC11946092; doi:10.3390/molecules30061214)
Supplement: Supplementary file 1 [file molecules-30-01214-s001.zip › molecules-3430751-supplementary.pdf]

Supplementary materials

Electrospun fibers from biobased and recycled materials for Indoor Air Quality enhancement

Natalia Czerwinska [1], Chiara Giosuè[1]*, Nicola Generosi[1], Mattia Pierpaoli[2], Rida Jbr[3], Francesca Luzi[1], Valeria Corinaldesi[1], Maria Letizia Ruello [1]

[1] Department of Science and Engineering of Matter, Environment and Urban Planning (SIMAU), Università Politecnica delle Marche, INSTM Research Unit, 60131 Ancona; N.C. n.czerwinska@staff.univpm.it, C.G. c.giosue@staff.univpm.it , N.G. n.generosi@staff.univpm.it, F.L. f.luzi@staff.univpm.it; V.C. v.corinaldesi@staff.univpm.it; M.L.R. m.l.ruello@univpm.it.
[2] Department of Metrology and Optoelectronics, Faculty of Electronics, Telecommunication and Informatics, Gdańsk University of Technology, Gdańsk 80-233, Poland; M.P. mattia.pierpaoli@pg.edu.pl
[3] Department of Civil, Environmental and Mechanical Engineering, University of Trento, Via Mesiano, 77,38123 Trento, Italy R.J. rida.jbr@unitn.it

*Correspondence: c.giosue@staff.univpm.it

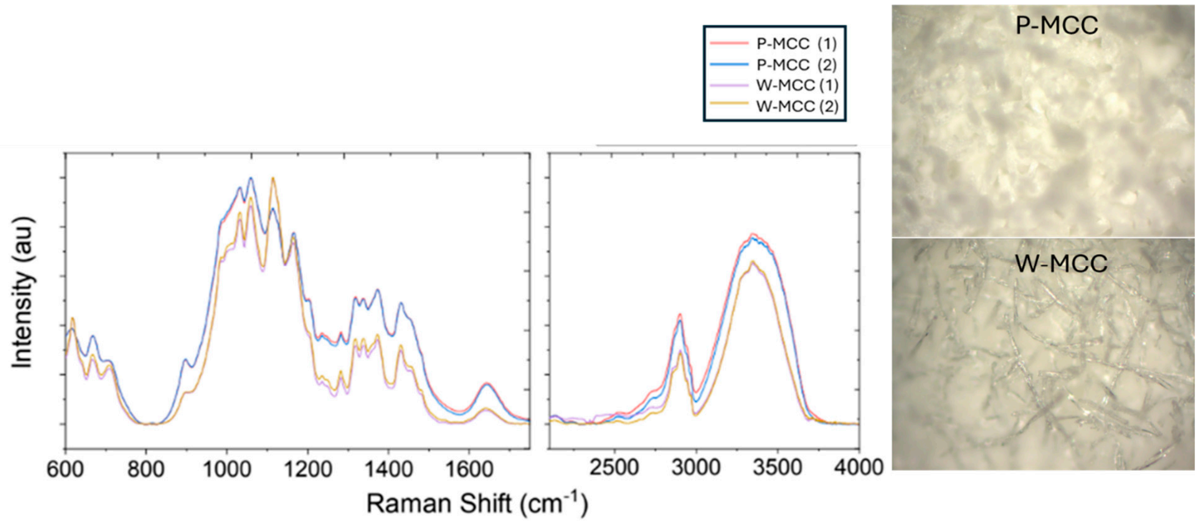

Figure S1. Raman spectra and images of P-MCC and W-MCC.

The P-MCC and W-MCC spectra are comparable. The only small difference is a more intense 1110 cm^{-1} peak in waste-derived microcrystalline cellulose (MCC) compared to pristine one, which is typically associated with C–O–C stretching vibrations of the glycosidic bonds linking glucose units in the cellulose

polymer. It can also correspond to C–O stretching vibrations in primary and secondary alcohol groups present in cellulose. However, the peak around 1110 cm^{-1} is most assigned to the C–O–C ether bond in cellulose.

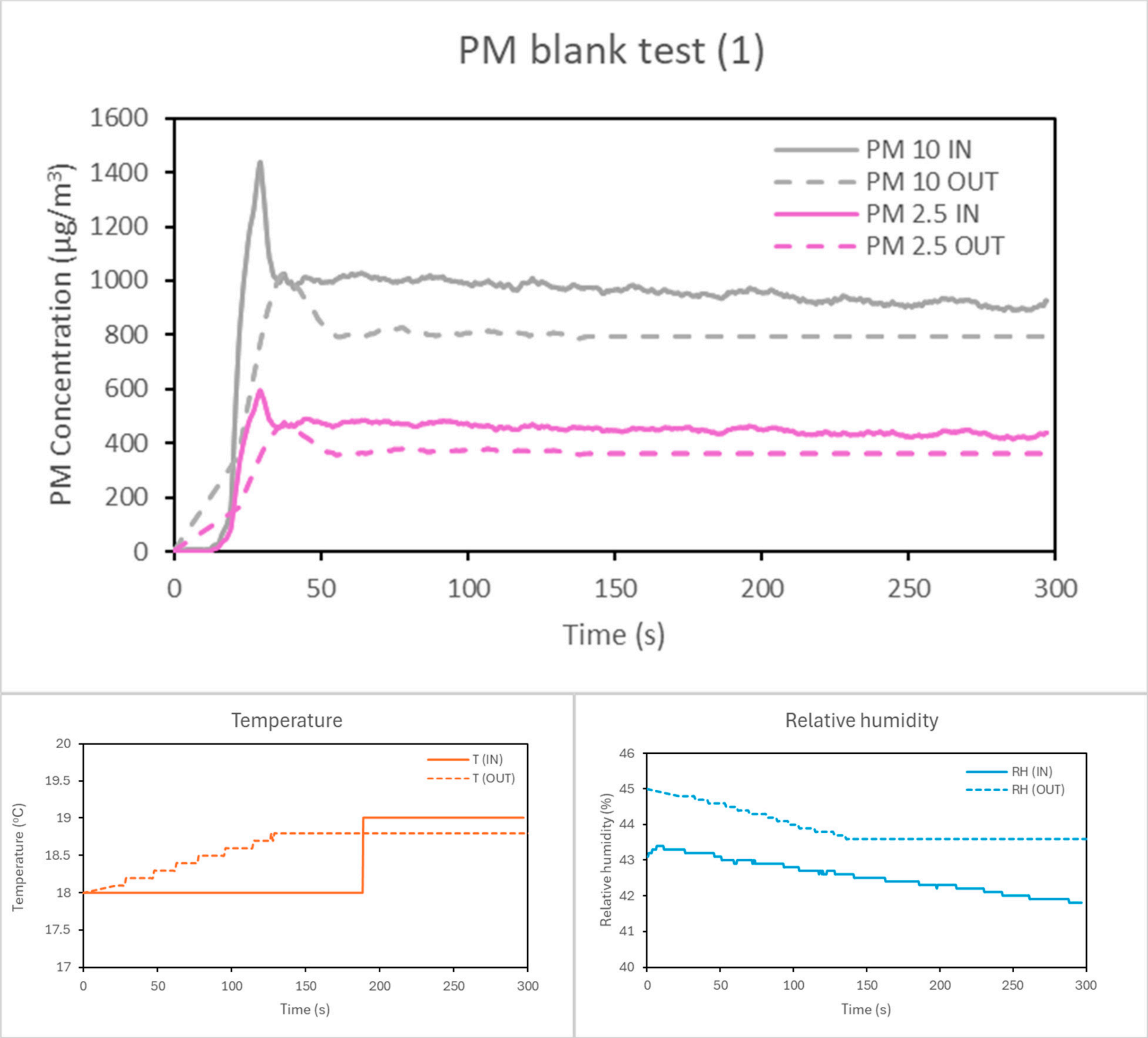

Figure S2. Calibration of PM filtration system (blank test 1).

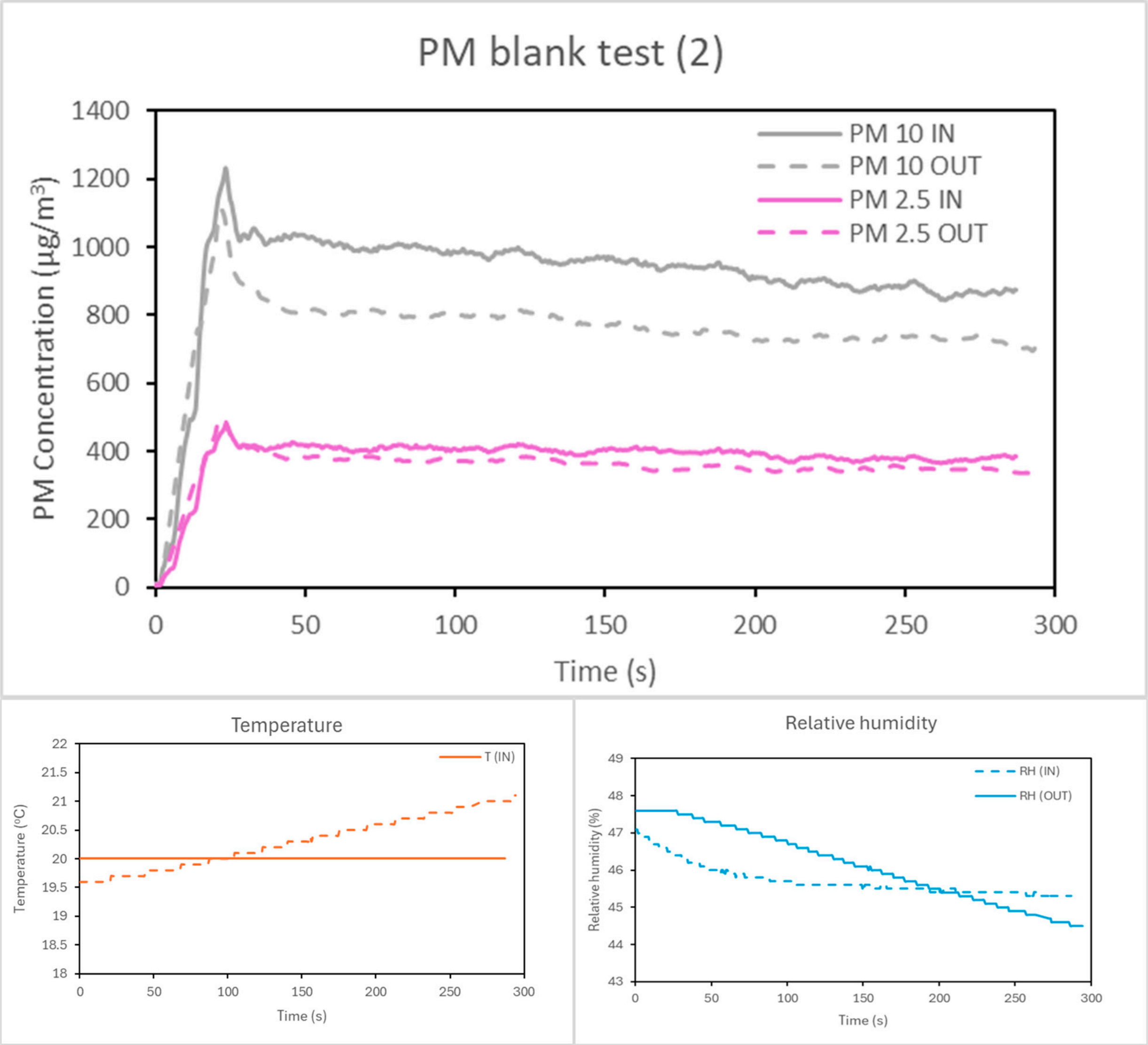

Figure S3. Calibration of PM filtration system (blank test 2).

Table S1. Results from calibration test.

	**$PM_{2.5}$ after 300s**		**Difference between IN and OUT**	**PM_{10} after 300s (μg/m³)**		**Difference between IN and OUT**	**Pressure drop**
	IN	OUT		IN	OUT		
Test 1	438 μg/m³	362 μg/m³	17%	925 μg/m³	796 μg/m³	13%	0 Pa
Test 2	384 μg/m³	334 μg/m³	14%	875 μg/m³	703 μg/m³	20%	0 Pa

The average difference in the concentration on the end of the test (after 300 s) between $PM_{2.5}$ (IN) and $PM_{2.5}$ (OUT) is 15 ± 3% and between PM_{10} (IN) and PM_{10} (OUT) is 17± 4%.

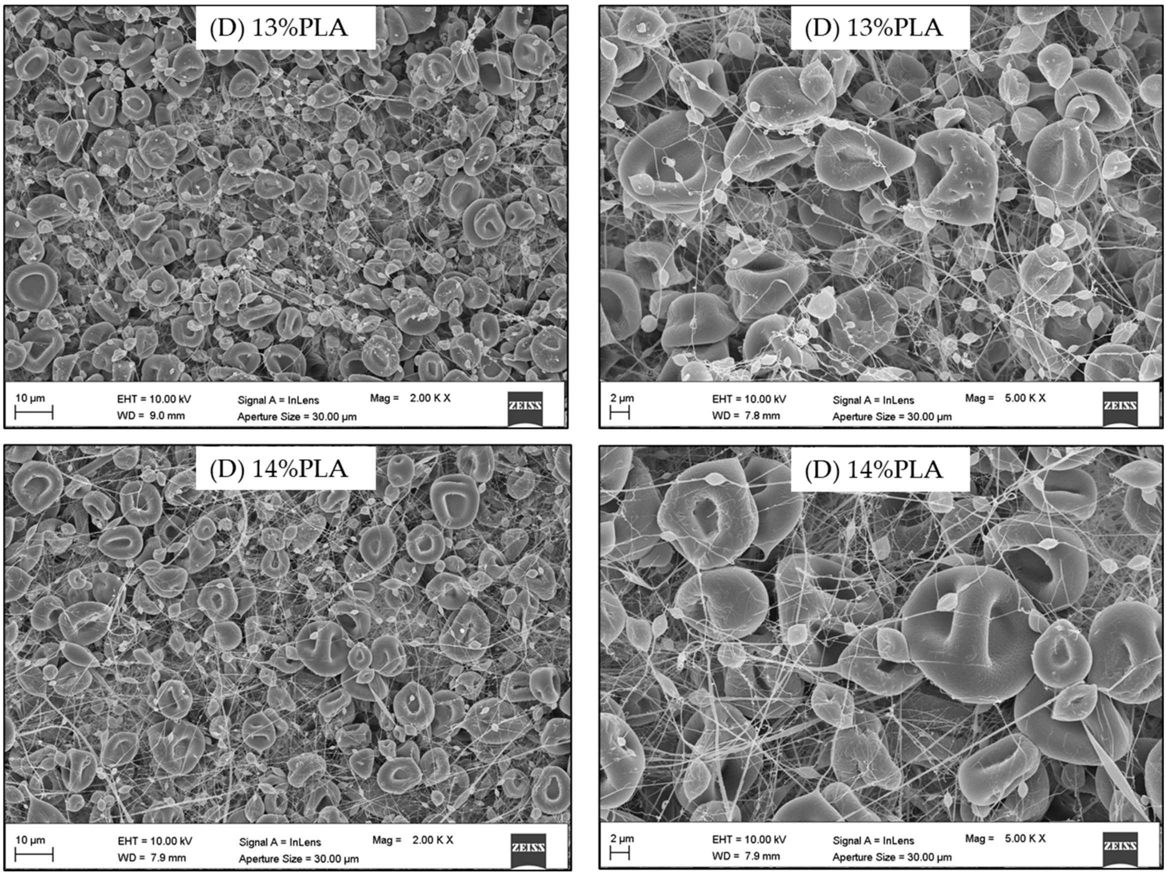

Figure S4. Morphological analysis of (D) 13%PLA and (D) 14%PLA samples.

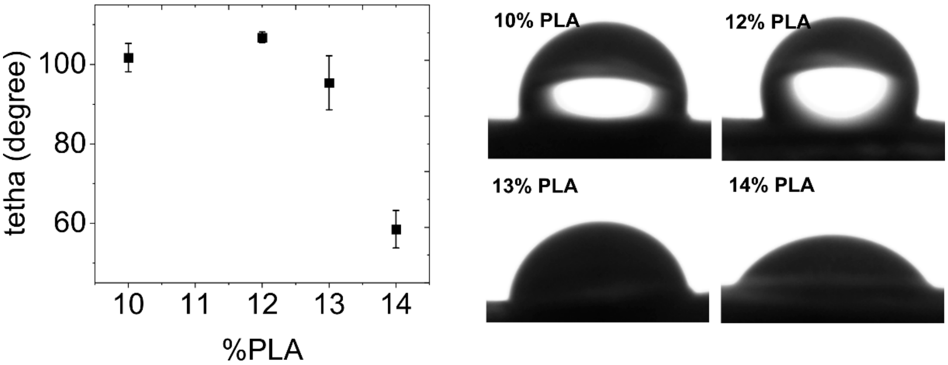

Figure S5. Comparison of the contact angle values for PLA fibers.

Table S2. Water contact angle results for (D) 13% PLA and (D) 14% PLA.

**Sample**	**Contact angle (°)**
(D) 13% PLA	95.4 ± 6.8
(D) 14% PLA	58.5 ± 4.7
